# Clearance of Apoptotic Cells by Tissue Epithelia: A Putative Role for Hepatocytes in Liver Efferocytosis

**DOI:** 10.3389/fimmu.2018.00044

**Published:** 2018-01-25

**Authors:** Scott P. Davies, Gary M. Reynolds, Zania Stamataki

**Affiliations:** ^1^Centre for Liver Research, College of Medical and Dental Sciences, Institute for Immunology and Immunotherapy, University of Birmingham, Birmingham, United Kingdom; ^2^Centre for Liver Research and National Institute for Health Research (NIHR) Birmingham Liver Biomedical Research Unit, College of Medical and Dental Sciences, Institute of Immunology and Immunotherapy, University of Birmingham, Birmingham, United Kingdom; ^3^University Hospitals Birmingham NHS Foundation Trust, Birmingham, United Kingdom

**Keywords:** efferocytosis, phagocytosis, liver, hepatocytes, regeneration, apoptosis, necrosis, cell death

## Abstract

Toxic substances and microbial or food-derived antigens continuously challenge the liver, which is tasked with their safe neutralization. This vital organ is also important for the removal of apoptotic immune cells during inflammation and has been previously described as a “graveyard” for dying lymphocytes. The clearance of apoptotic and necrotic cells is known as efferocytosis and is a critical liver function to maintain tissue homeostasis. Much of the research into this form of immunological control has focused on Kupffer cells, the liver-resident macrophages. However, hepatocytes (and other liver resident cells) are competent efferocytes and comprise 80% of the liver mass. Little is known regarding the mechanisms of apoptotic and necrotic cell capture by epithelia, which lack key receptors that mediate phagocytosis in macrophages. Herein, we discuss recent developments that increased our understanding of efferocytosis in tissues, with a special focus on the liver parenchyma. We discuss the impact of efferocytosis in health and in inflammation, highlighting the role of phagocytic epithelia.

## Key Points

- Efferocytosis is a vital process in tissues that can be carried out by multiple cell types, including blood derived and tissue resident phagocytes.
- Hepatocytes are competent efferocytes and play an important role in the clearance of dead cells in health and in inflammation.
- Epithelial cell efferocytosis is understudied and involves distinct mechanisms to professional phagocytes.
- Defects in efferocytosis have been linked to diseases such as autoimmunity, failure to prevent metastasis, failure to limit infection.
- Understanding molecular mechanisms of efferocytosis may reveal new pathways for therapeutic intervention to alleviate inflammation.

## Efferocytosis in the Liver

Efferocytosis, the clearance of dead and dying cells, is important to prevent tissue damage and promote the resolution of inflammation (1). The liver has evolved into an expert in defusing biochemical threats emanating from food or microbial antigens, which reach the organ along with 75% of its blood supply that arrives through venous blood from the gut. Hepatocytes comprise 80% of liver cells and constitute the biochemical powerhouses of the liver parenchyma, and as a result they often perish in their duties to absorb toxic substances. To cope with loss of hepatic epithelia, the liver has evolved the remarkable ability to regenerate.

**Figure 1:**
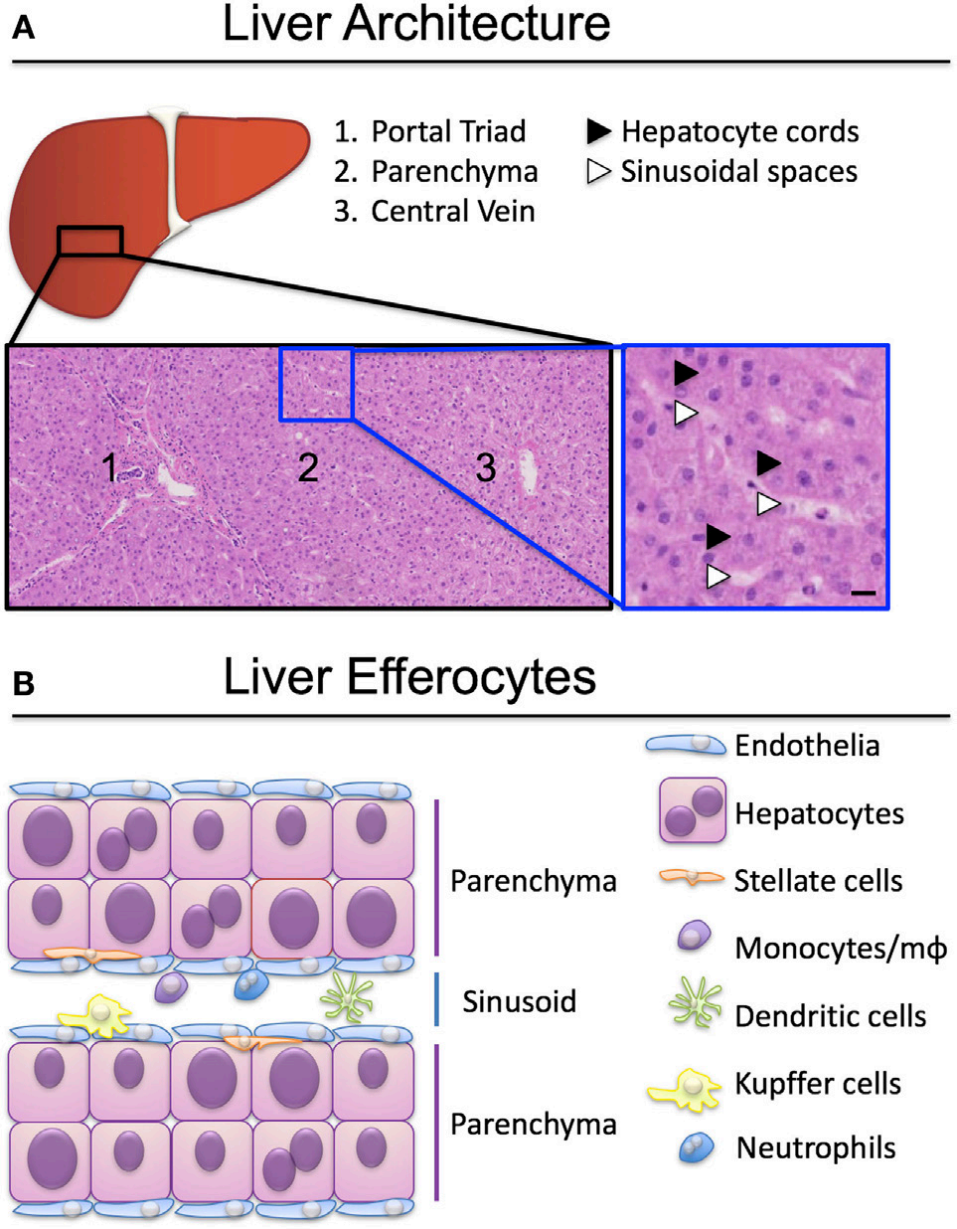
Organization of liver-resident and recirculating efferocytes. **(A)** Hepatocytes are spread over three zones, exposed to different levels of oxygen and nutrients. Hepatocytes in zone 1 proximal to the portal triad (portal vein, hepatic artery, bile duct) have access to arterial and venous blood entering the liver through the circulation. Hepatocytes in zone 3 have less access to oxygen and nutrients and are exposed to blood draining into the central vein. Hematoxylin-eosin stain, scale bar represents 50 µm. **(B)** A plethora of liver resident and recirculating cells are able to engulf apoptotic and necrotic cells and clear them to maintain tissue homeostasis. Kupffer cells, monocytes and macrophages (mϕ) are the best-characterized efferocytes in the liver.

To perform their detoxification roles, hepatocytes are strategically organized roughly into two hepatocyte-thick cords, flanked by a thin layer of fenestrated endothelia (Figure 1). Nutrient-rich blood enters the liver *via* the portal vein and oxygen-rich blood *via* the hepatic artery, which, together with a bile duct, form the liver portal triad (Figure 1A). Blood from both sources mixes in the specialized hepatic capillaries termed sinusoids, and drains toward the central vein. Hepatocytes near the portal triads (designated zone 1) can be damaged by the inflammatory infiltrate during interface hepatitis, when immune cells cross the sinusoidal endothelia and reach the parenchyma. Zone 2 is found mid-distance from a portal triad and the draining central vein (zone 3). Periportal hepatocytes near zone 1 have access to oxygenated blood from the hepatic artery, and nutrients from the portal blood supply that arrives from the gut. Oxygen and nutrient levels reduce toward the central vein and hepatocytes in zone 3 are found in hypoxic conditions. Fenestrations in the sinusoids allow hepatocytes access to solutes and immune cells reaching through the fenestrations from the circulation (2, 3), but prevent unregulated migration of immune cells to the parenchyma (4).

Cells that perish in the sinusoidal spaces are cleared by circulating phagocytes (monocytes, dendritic cells, neutrophils), liver-resident macrophages termed Kupffer cells, and by sinusoidal endothelia (Figure 1B). The best-characterized liver efferocytes are macrophages, both those derived from monocytes infiltrating from the circulation, and the self-renewing populations of Kupffer cells. It is understood that professional phagocytes are activated during injury and adapt their phenotype following the encounter of cellular debris, danger signals, and soluble mediators of the inflammatory milieu. The critical role of liver macrophages including Kupffer cells in the ebb and flow of inflammation was recently reviewed by Tacke’s group (5, 6).

Activated hepatic stellate cells can also engulf apoptotic hepatocytes, which in turn lead to increases in tumor growth factor-β (TGF-β) secretion (7). Biliary epithelial cells (BECs) also take part in efferocytosis of neighboring apoptotic cells; an important adaptation for diseases associated with increased BEC apoptosis such as primary biliary cholangitis (8). The phagocytic activity of hepatocytes was noted in 1992 (9). Hepatocyte efferocytosis assists in parenchymal housekeeping to rapidly dispose of cell remnants and prevent excessive inflammation.

**Figure 2:**
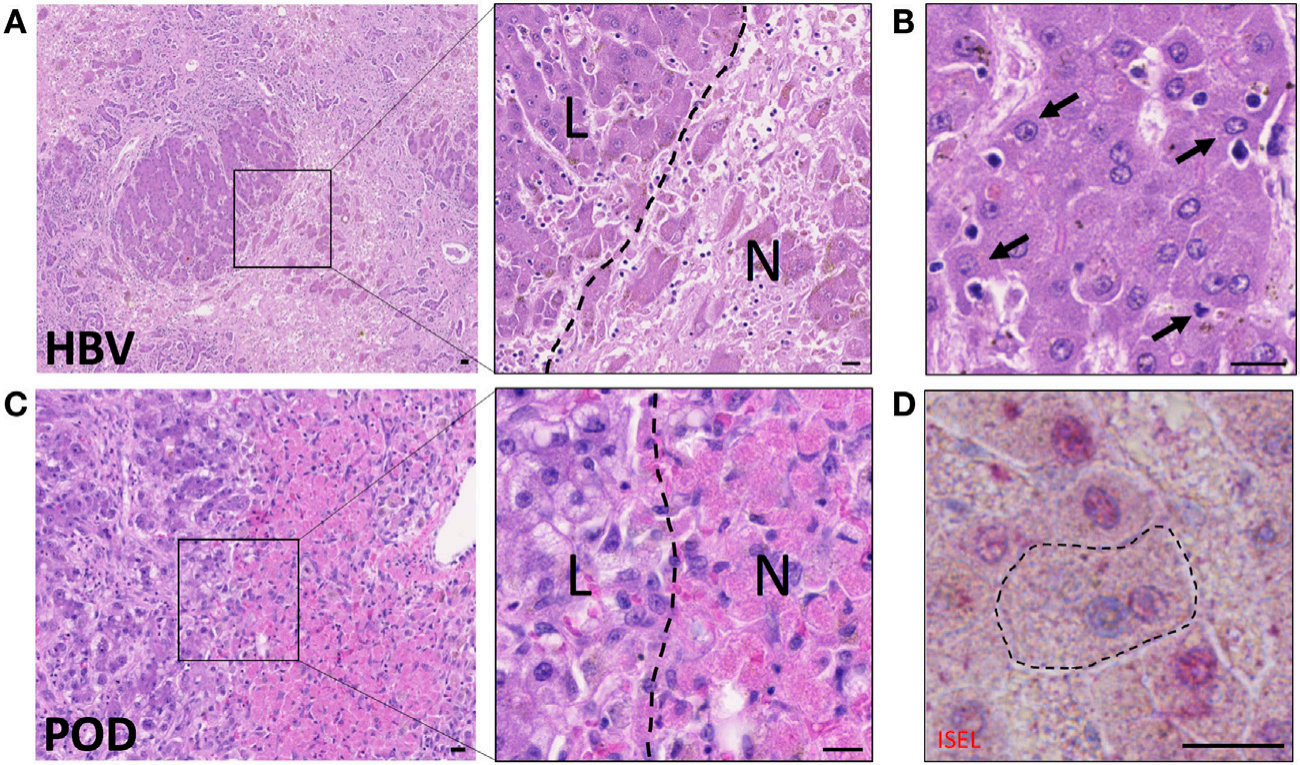
Hepatocytes engulf necrotic and apoptotic cells in acute-on-chronic liver injury caused by hepatitis B infection (HBV) and in paracetamol injury (POD). **(A)** Hematoxylin–eosin staining of acute-on-chronic liver injury in a patient with HBV infection. Large areas of hepatocyte necrosis are evident. Inset image shows dark stained hepatocyte nuclei in live hepatocytes (L) and pyknotic or karyolytic nuclei in necrotic hepatocytes (N). **(B)** Healthy hepatocytes with clearly marked nuclei are seen phagocytosing small apoptotic cells (arrows). Note hepatocyte invaginations which have formed to enable capture of apoptotic cells. **(C)** Hematoxylin-eosin staining of liver with paracetamol-induced injury, which causes centrilobular necrosis. Inset shows pink cytoplasm in necrotic hepatocytes (N) compared to surviving non-discolored hepatocytes with clearly defined nuclei (L). **(D)** *In situ* end labeling (ISEL) of apoptotic cell nuclei is seen here in pink, in a liver with ischemia-reperfusion injury. The marked hepatocyte has a non-apoptotic nucleus seen in blue, and has engulfed an apoptotic cell with a pink nucleus. Neighboring apoptotic hepatocytes can be seen with pink nuclei, and non-apoptotic cells with blue nuclei. The bars show 20 µm.

Hepatocyte death from biochemical toxicity (*necrosis*) occurs in health as part of normal homeostasis, however, liver damage is exacerbated in infection or in alcoholic, drug or ischemia-induced liver injury where large areas of necrotic lesions are evident (Figure 2). Acute-on-chronic liver failure is a syndrome associated with exacerbation of hepatitis B infection (HBV) and characterized by broad areas of hepatic necrosis in cirrhotic patients (Figure 2A). Lymphocyte infiltration is often seen in the parenchyma in chronic liver diseases. Crispe and others have elegantly put forward the “graveyard theory” where the liver is primary site for the disposal of spent immune cells (10). Figure 2B shows hepatic epithelia in the process of engulfing immune cells that have perished in the parenchyma, and this is seen predominantly near the portal regions. Conversely, in cases of acute liver injury such as paracetamol overdose (POD), hepatocyte necrosis due to loss of ATP is noted around the centrilobular regions (zone 3, Figure 2C). Histological features of necrotic hepatocytes include eosinophilic degradation and pyknotic nuclei, which are readily detectable by hematoxylin-eosin staining (inset, Figure 2C).

Hepatocytes also clear away cells that have triggered the molecular cascade of events of programmed cell death (*apoptosis*) (Figures 2B,D), but can actively destroy live autoreactive immune cells by direct engulfment as noted for CD8^+^ T cells undergoing *suicidal emperipolesis* (11). Immune cell death and liver damage are exacerbated in chronic liver inflammation of multiple etiologies, including autoimmune, metabolic, viral, and genetic diseases (12, 13). The rapid processing of dead and dying cells is vital to moderate inflammation (12, 14, 15).

It is remarkable how little we know about the molecular mechanisms that govern the ability of the largest internal organ in the body to mediate the clearance of damaged or dying cells, given that this is one of the liver’s major functions. Herein, we bring together research on hepatocyte efferocytosis and place it into context with current molecular knowledge on the clearance of dead cells by immune phagocytes.

## Clearance of Apoptotic and Necrotic Cells

Cells die through a wide array of processes, each situational and requiring their own dedicated cascade of signaling events. The most frequent forms of cell death are attributed to apoptosis or necrosis. Apoptosis, an active form of programmed cell death, is characterized by the initiation of specific inducible pathways (16, 17). This includes the extrinsic pathway; the engagement of extracellular signals, including Fas ligand (FasL) (18) and tumor necrosis factor family cytokines (TNF) (19), amongst others, to their respective death receptors which initiates intracellular death signaling. Apoptosis can also be triggered intrinsically; certain signals, such as a lack of growth factors, endoplasmic reticulum stress or DNA damage, can induce a shift in expression of Bcl-2 family mitochondrial proteins (20). Increased activity of proapoptotic proteins lead to cytochrome C release and caspase 9 activation. Apoptosis pathways result in the activation of effector caspases (3, 6, and 7), which in turn begin to proteolytically degrade the cell’s components. Apoptotic cells are generally smaller than live cells and can be identified by the formation of surface blebs (16).

Necrosis is considered a passive, unprogrammed type of cell death and is often incurred accidentally, although active mechanisms of necrosis have also been reported (21). While multiple mechanisms can induce necrosis, the major causes are attributed to compromising of the plasma membrane, or depletion of energy (22). Furthermore, apoptotic cells can be converted to necrotic cells (also known as *secondary necrosis*) if ATP levels fall below the quantity required to complete the active apoptotic process (23). The appearance of necrotic cells is often swollen with disrupted organelle and plasma membranes (24). The nucleus is often broken down and will be unstained by hematoxylin (Figure 2). As necrosis often occurs in areas of tissues, rather than the single cell death hallmark of apoptosis, often multiple necrotic cells can be identified in one area. Due to their lack of integrity, necrotic cells will often form cell debris, which can induce liver damage if not cleared swiftly, as we discuss in later sections.

Upon the death of a cell, its corpse must be cleared through efferocytosis. This is a specialist form of phagocytosis, whereby fragments of the dying cells are engulfed by other cells, which in turn degrade and recycle their components. Although both apoptotic and necrotic cells are often captured by the same efferocytes, each are recognized through different means and yield differing response in the predatory cell (25). Apoptotic cells are most commonly recognized through the display of the phospholipid phosphatidylserine (PtdSer) on the outer leaf of the plasma membrane that can be recognized by many receptors [phosphatidylserine receptors (PSRs)] directly (26) or *via* association with low-density lipoprotein (27, 28). Of note, in a rat liver model, it was shown that recognition of apoptotic cells from mice or humans was reduced compared to rat cells; it is therefore possible that species-specific recognition molecules can mediate efferocytosis (29).

A phenotypic aspect of apoptotic cells is that, although shriveled, the cell remains intact as a singular body. This allows for a clean removal of the dying cells by efferocytes, usually without provoking an inflammatory response. How intact a necrotic cell remains is reflected through the manner in which cell death was induced. As such, multiple modalities for necrotic cell recognition are necessary to guarantee their clearance. Some reports have suggested that necrotic cells can also be recognized by PSRs (30). However, due to the lack of integrity of most necrotic cells, they are often recognized through molecules exposed by necrotic death (25). The same mechanisms are also used to detect pathogens. For example, complement receptors and Fc receptors detect opsonized necrotic cells, and this recognition can trigger signaling events that activate the phagocyte (31–35). As such, necrotic cells are engulfed through the detection of autoantigens, which often increases the risk for autoimmune disease. Necrotic cells can also be indirectly recognized through opsonin engagement of other cellular components. For example, ficolin-2 and -3 have been shown to bind DNA, facilitating the clearance of late-apoptotic/necrotic cells through interactions with calreticulin (33, 36). A ubiquitous mechanism for clearance of necrotic cells remains uncertain.

The differences between recognition, and thus further downstream signaling of apoptotic and necrotic cells, result in conversing consequences for the efferocyte (37). Apoptotic cell clearance generally leads to the production of anti-inflammatory stimuli and pro-resolution signals for inflammation such as interleukin 10 (IL-10) and TGF-β (38). Conversely, necrotic clearance generally results in pro-inflammatory signaling, as many of the recognition receptors are also required for pathogen recognition. In the liver, the signals associated with hepatocyte death were recently reviewed by Brenner and colleagues (39). In this work the importance of the extent and duration of dead cell accumulation was highlighted, as mild and localized cell death can aid regeneration and exert hepatoprotective effects. Equally, prolonged and wide-spread cell death can exacerbate liver injury.

## Experimental Systems to Study Efferocytosis

**Figure 3:**
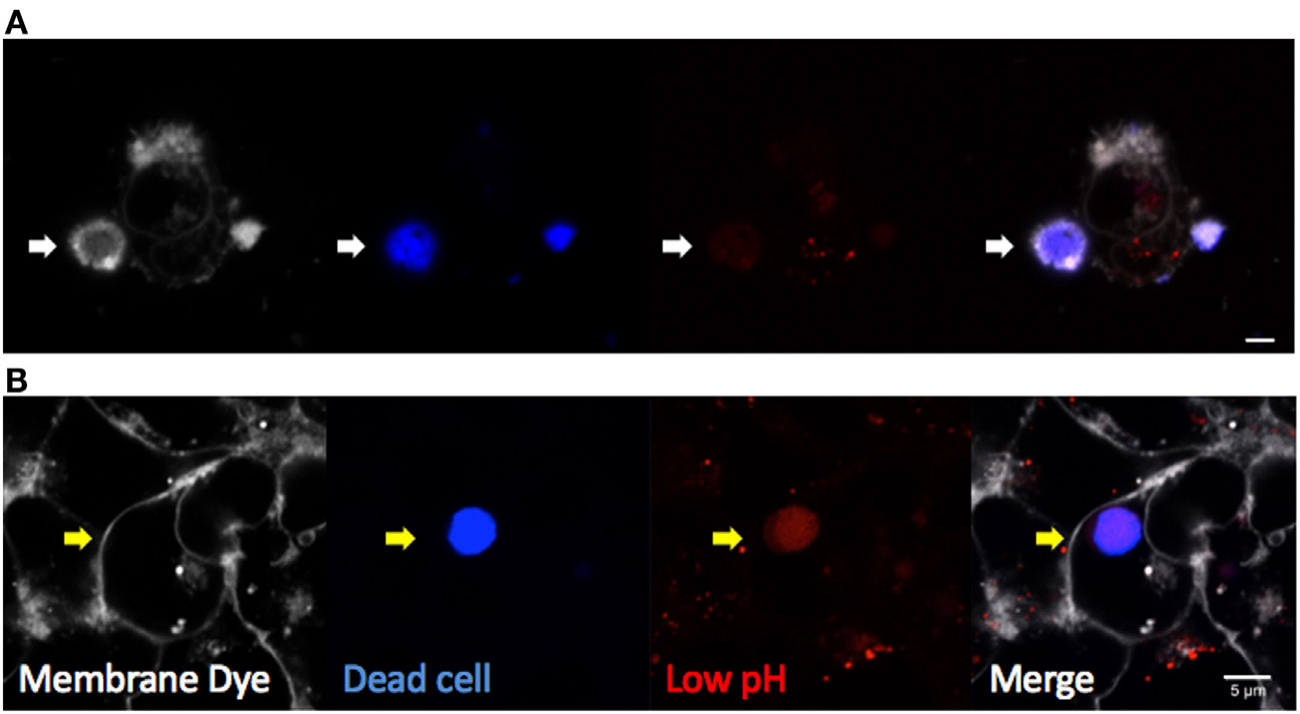
Visualizing efferocytosis by confocal microscopy. Hepatic epithelia were cocultured with violet-labeled staurosporin-treated apoptotic Jurkat T cells in the presence of pHrodo red, which only fluoresces in conditions of low pH (Thermo Fisher Scientific). CellMask Plasma Membrane stain was added to the culture media to label all exposed cell membranes before imaging. **(A)** Non-internalized apoptotic cells (blue) attached to hepatocytes were labeled by CellMask Plasma Membrane in white, and they were not labeled by pHrodo red dye (white arrow). **(B)** Internalized dead cells were not accessible to the membrane dye, confirming internalization (yellow arrow). Complete internalization into an acidic compartment was confirmed by pHrodo red, which detected efferosome acidification as early as 3 hours following engulfment. The scale bar indicates 5 µm.

Multiple techniques have been described for both *in vivo* and *in vitro* studies of efferocytosis. Fluorescent dye-labeled efferocytes can be “fed” alternatively labeled dead cells under varying conditions and time courses. Early apoptosis can be confirmed by Annexin V labeling of the cell surface as it binds directly to PtdSer, although care must be taken when studying certain activated cell types or using calcium-sensitive protocols (40). Later stages of apoptosis or necrosis are often confirmed with cell impermeable DNA dyes such as 7AAD or TOPRO-3 iodide, which can enter cells once the membrane is compromised. Combined labeling with Annexin V and a membrane-impermeable DNA label was developed to identify the stages of apoptotic cells in more detail (41). Cells can then be assessed by flow cytometry, or imaged by fluorescent microscopy. Complete internalization of dead cells can be confirmed by lack of access to membrane dyes added to the culture media (such as CellMask Plasma Membrane Stains, Thermo Fisher Scientific) or demonstration of efferosome acidification using pH indicator dyes (Figure 3). Quantitative analyses by confocal and time-lapse microscopy can be useful to determine the frequency and kinetic of efferocytosis *in vitro*.

Fluorescent labeling of dead cells and efferocytes may also be adapted for flow cytometry-based studies, whereby double-positive cells represent efferocytes containing cargo. This form of analysis has been used to study the clearance of neuraminidase-treated red blood cells in mice (42). Other studies opt to analyze efferocytosis using downstream secreted molecules as proxy to utilize alternative techniques such as reporter assays. The capacity of Scavenger Receptor Class F Member 1 (SCARF1) to act as a dead-cell receptor on transfected HEK293T cells, for example, was confirmed using IL-8 mRNA production as a marker of NF-κB activation following apoptotic and necrotic cell efferocytosis (43).

Efferocytosis is not often as straightforward to detect *in vivo*. Fluorescent labeling can enable temporal measurements in mouse models by intravital imaging of the liver (44), but the technique remains to be adapted successfully for use in human tissues *ex vivo*. Molecular markers of cell death for use with fixed tissue are often important for the confirmation of efferocytosis. Caspase 3/7 activation or their effects can be measured to delineate apoptotic bodies by immunohistochemistry (IHC) or immunofluorescence (IF) (45). DNA end-labeling is frequently used to confirm the death of cells in tissues. End-labeling involves the addition of labeled nucleotides to DNA breaks induced throughout multiple modalities of death, using a DNA polymerase. This was historically used for *in situ* end labeling (ISEL) of fixed tissue sections as part of IHC chromagen staining (Figure 2D) (46). This was then adapted for the creation of terminal deoxynucleotidyl transferase (TdT) dUTP Nick-End Labeling (TUNEL) (47) which substitutes a polymerase for TdT. This adaptation allows for the use of many different modified forms of labeled nucleotides (often dUTP), such as non-reactive protein tags or fluorophores. TUNEL staining has been altered to specifically identify cells in late-stage apoptosis.

Further stains for cell membrane proteins or specific nucleic proteins can be used to determine complete engulfment of dead cells. Many of these techniques were exemplified in a recent study of macrophage/monocyte efferocytosis in models of acute liver injury (48). In this work, Antoniades and colleagues studied the mechanism of resolution of liver inflammation through apoptotic cell clearance by macrophages/monocytes *via* Mer tyrosine kinase receptor (MerTK). Staining for myeloperoxidase (activated neutrophils) combined with TUNEL allowed for the identification of apoptotic neutrophils in human liver, both through IHC and IF staining. Additionally, fluorescent monocytes were cocultured *in vitro* with alternatively fluorescent apoptotic hepatic cells or neutrophils. The ability of these monocytes to clear apoptotic cells was then assessed through fluorescent microscopy and flow cytometry. Both techniques were used to show the increased capacity of monocytes for dead cell clearance following stimulation with secretory leukocyte protease inhibitor.

## The Mechanism of Dead Cell Capture by Professional and Non-Professional Efferocytes

Phagocytes express several receptors to recognize and subsequently clear dying cells from the tissues (49–51). In the case of professional phagocytes (e.g., macrophages) multiple apoptotic and necrotic cell receptors have been characterized and these remain relevant in the liver (37, 52) (Table 1). First described in 1992, it is now widely accepted that apoptotic cells are recognized through their expression of PtdSer on the outer leaf of the plasma membrane (53, 54). Several receptors directly recognize PtdSer, many of which are expressed by professional phagocytes (55). These include stabilin-1, stabilin-2, brain-specific angiogenesis inhibitor 1 (BAI1), and RAGE, as well as the TIM family of transmembrane glycoproteins, including TIM-1, -3, and -4 (56–61). Mammary, alveolar and mesangial epithelia recognize apoptotic cells *via* the PSR, CD36, the vitronectin receptor αvβ3, and CD91 (62–64). Of note, molecules that bind PtdSer such as high-mobility group box 1 (HMGB1) can also downregulate apoptotic cell clearance (65, 66).

**Table 1:** Efferocytosis receptors in professional phagocytes and tissue epithelia.

Name	Cell type	Target	Reference
**Professional phagocyte receptors (macrophages/dendritic cells)**			

αvβ3 integrins	Macrophages	Lactadherin (MFG-E8)—PtdSer, vitronectin	(67–69)
αvβ5 integrins	Macrophages	Lactadherin (MFG-E8)—PtdSer, vitronectin	(67, 69–72)
	Dendritic cells		
Axl	Monocytes/macrophages	Gas6-PtdSer, Tubby-like protein 1 (GULP), Protein S	(73–76)
BAI1 (brain-specific angiogenesis inhibitor 1)	Macrophages	PtdSer	(77)
Calrecticulin/CD91	Monocytes, macrophages, neutrophils	Complement component C1q	(34)
CD11/b/c/CD18	Monocytes, macrophages, neutrophils, human DCs	Complement component C3bi	(78, 79)
CD14	Macrophages	Phospholipids (not PtdSer-dependent)	(80, 81)
CD36	Macrophages	Thrombospondin + PtdSer + oxLDLs	(82–84)
Clec9a	Dendritic cells	Necrotic cells, exposed actin filaments	(85, 86)
LOX1	Macrophages	oxLDLs—PtdSer	(28, 87)
MARCO	Macrophages	Uncertain	(88)
MerTK	Monocytes/macrophages	Gas6-PtdSer, Tubby, Protein S, Tubby-like protein 1, Protein S	(75, 89, 90)
Phosphatidylserine receptor (PSR)	Monocytes/macrophages	PtdSer	(91)
RAGE (receptor for advanced glycation end products)	Alveolar macrophages	PtdSer	(60)
SCARF1	Monocytes and dendritic cells	Complement component C1q—PtdSer	(43)
Scavenger receptor A (SR-A)	Macrophages	Uncertain	(88)
SIGN-R1 (specific intercellular adhesion molecule-3-grabbing nonintegrin-related 1) (murine)	Mouse marginal zone macrophages	Not confirmed for apoptotic cells	(92)
Stabilin-1 (CLEVER-1)	Tissue-specific, alternatively activated macrophages	PtdSer	(61)
Stabilin-2	Macrophages	PtdSer	(57)
TIM-3 (T cell/transmembrane, immunoglobulin, and mucin 3)	Dendritic cells	PtdSer	(93)
TIM-4	Monocytes/macrophages	PtdSer	(94)
Tyro3 (sky)	Monocytes/macrophages	Gas6-PtdSer, Protein S, Tubby-like protein 1, Protein S	(73–76)

**Non-professional phagocytes**			

αvβ5 integrins	Retinal epithelial cells	Lactadherin (MFG-E8)-PtdSer	(95)
ASGPR (asialoglycoprotein receptor)	Hepatocytes	asialoglycoprotein	(96, 97)
CD36	Retinal Pigment cells	PtdSer	(98)
KIM-1 (kidney injury molecule 1)/TIM1 T cell/transmembrane, immunoglobulin, and mucin 1	Injured kidney endothelial cells	PtdSer	(99)
LOX1	Endothelial cells	oxLDLs—PtdSer. Ca^2+^-dependent	(28, 100)
MerTK	Retinal pigment cells	Gas6-PtdSer, Tubby, Protein S, Tubby-like protein	(89, 90)
Phosphatidylserine receptor (PSR)	Fibroblasts	PtdSer	(91)
	Epithelial cells		
	T and B lymphocytes (ectopic expression)		
SCARF-1 (SREC-1)	Endothelial cells	Complement component C1q—PtdSer	(43)
Stabilin-1 (Clever-1)	Human sinusoidal endothelial cells	PtdSer	(101)
Stabilin-2	Human sinusoidal endothelial cells	PtdSer	(101)

It is common for PtdSer to be recognized in complex with certain bridging molecules. Some of the most well-studied PtdSer receptors, the TAM tyrosine kinases (Tyro3, Axl, and MerTK) work in this manner (102); notably, hepatocytes express Axl but not Tyro3 or MerTK (103). The earliest known examples of these are Gas6 and Protein S (104, 105). Gas6 is universally recognized by TAM receptors, whereas Protein S, which is expressed in hepatocytes, is not recognized by Axl. Similarly, integrins αvβ3 and αvβ5 have been shown to promote efferocytosis through the recognition of PtdSer in complex with lactadherin, also known as milk fat globule EGF factor 8 (MFG-E8) (67, 68, 106).

The entirety of apoptotic cell recognition does not lie with the detection of PtdSer expression. It was shown that Tubby protein and its relative Tubby-like protein 1 (TuLP1), which do not bind PtdSer, specifically localize at the surface of apoptotic cells and could act as TAM receptor bridging molecules in a similar manner to Gas6, which in turn promoted apoptotic cell clearance (89). All TAM tyrosine kinases recognized TuLP1, whereas Tubby was exclusively recognized by MerTK on macrophages and retinal pigment cells. Mechanisms of immune surveillance and signaling have also been shown to contribute to apoptotic cell clearance. Components of the complement pathway have been shown to induce phagocytosis in macrophages and DCs by opsonizing apoptotic cells, including C1q and C3 (34, 78, 107). Furthermore, SIGN-R1, a mouse analog of human mannose receptor DC-SIGN, was shown to bind apoptotic cells and induce their labeling with C3 and subsequent clearance by marginal zone macrophages (92).

Recognition of apoptotic cells, although important, is not sufficient for macrophages to engulf and clear them. Downstream intracellular signaling is necessary for load-processing following capture. An important, highly-conserved signaling pathway has been described downstream from most common PtdSer-receptors, involving GTPase Rac1 and ELMO1-DOCK180 interactions (50). TAM-family molecules, αvβ5 integrins and BAI1 act as docks for apoptotic cells, leading to intracellular signaling *via* this pathway (108, 109). Upon engagement of an apoptotic cell by these receptors, DOCK180 is recruited by ELMO1 (110, 111). In complex, these proteins act as guanine exchange factors, allowing for Rac1 activation, which induces necessary cytoskeletal arrangements required for complete engulfment of the prey cell. Stabilin 1 and 2 have also been shown to activate this pathway through the adaptor protein GULP (109, 112, 113). Completion of apoptotic cell engulfment also commonly involves the activation of nuclear receptors. Loose nucleotides released from dying cells commonly act as “*eat-me*” signals, and can engage purigenic P2 receptors (P2X and P2Y), leading to an increased capacity for efferocytosis in macrophages (114, 115). It was recently shown that liver X receptor (LXR) was necessary for the capture and processing of apoptotic cells by macrophages and dendritic cells (116, 117). LXR responds to oxysterols found in engulfed apoptotic cells. Stimulation of LXR upregulated MerTK and anti-inflammatory cytokines IL-10 and TGF-β, while also leading to the downregulation of proinflammatory cytokines such as IL-1β, CCL2, and MARCO. A-Gonzalez and Hidalgo reviewed nuclear receptors and their role in macrophage efferocytosis recently (118). LXRα mediates fatty acid regulation in hepatocytes (119), but its role in hepatocyte efferocytosis remains to be determined.

Non-professional phagocytes, such as epithelial cells express multifunctional scavenger receptors, or molecules that exert alternative functions in other cell types. For example, TIM-1, also known as kidney injury molecule 1 (KIM-1), is known to possess multiple immune functions, including CD4^+^ T-cell and mast cell activation (59). However, TIM-1 was also upregulated in kidney epithelia following injury, allowing for a temporary efferocytic capability (99). Certain cell-exclusive receptors and modulators associated with apoptotic cell clearance have also been described. Apoptotic cell clearance in the liver has been shown to involve asialoglycoprotein receptor (ASGPR) on hepatocytes (96). This ASGPR1 and ASGPR2 complex is critical for receptor-mediated endocytosis of terminally desialylated glycoproteins and is restricted to the liver. Autoantibodies to ASGPR have been found in patients and models of autoimmune hepatitis (120–122). Resolvin D1 is also important in liver protection from ischemia/reperfusion injury, by enhancing efferocytosis by M2-polarizing macrophages (123). Furthermore, phagocytosis by retinal pigment cells, although mostly conducted through MerTK, was shown to be increased through recognition of ATP-binding cassette subfamily F member 1 (ABCF1) released from apoptotic photoreceptor outer segments (124). Overall, it appears that although the broad function of recognition and engulfment of apoptotic cells is conserved throughout many cell types, multiple mechanisms exist that conduct these processes across phagocytes, both homo- and heterotypically.

## Regulation of Efferocytosis

External stimuli are pertinent for regulation of dead cell clearance by efferocytosis. As such, “*find-me*” signals released by apoptotic cells are often necessary for the guidance of efferocytes to their prey (53). The best-characterized examples of these are extracellular nucleotides (115). It was shown that upon caspase 3/7 activation in apoptotic cells, ATP and UTP released from apoptotic cells could recruit monocytes/macrophages through recognition by P2Y_2_. Conversely, molecules with the opposite effect known as “*don’t eat-me*” signals have also been described. CD47 is the most notable, having been shown to provide resistance to clearance by macrophages on malignant cells and more recently on atherosclerotic plaques (125, 126). Similar “find-me” signals may be utilized by non-motile phagocytes, which extend protrusions to collect apoptotic cells for clearance but are restricted to targets within their tissue niche.

Due to the influence of dying cells on the immune response, cytokine and growth factor stimulation of both professional and non-professional phagocytes can regulate their capacity to clear dead cells. Apoptotic T-cell lymphomas release sphingosine-1-phosphate, a bioactive lipid often involved in immune cell recruitment, leading to the recruitment of macrophages and monocytes (127). Similarly, certain chemokines, tasked with immune cell recruitment have also been shown to increase phagocyte recruitment to areas of apoptotic cells. CX_3_CL1 (fractalkine) was shown to recruit macrophages to its source, apoptotic Burkitt lymphoma cells (128).

Multiple cytokines have varying effects on efferocytosis (129). Most notably, secretion of IL-3 and IL-14 increased efferocytosis in macrophages through activation of PPAR and increase in CD36 expression (130, 131). IL-4 has been reported to upregulate expression of other PtdSer-receptors such as stabilin 1and 2 (61). IL-10 and TGF-β can also increase efferocytes by macrophages (132, 133). In contrast, pro-inflammatory cytokines reduce the capacity for dead cell engulfment: TNF-α has been shown to inhibit efferocytosis in macrophages (134) and both IFN-γ secretion and receptiveness were reversely correlated with anti-inflammatory cytokines and receptors including IL-4 and TIM receptors (59, 129, 135). However, this was not always the case for these cytokines. Both TNF-α and IFN-γ have been shown to increase LOX-1, which may recognize apoptotic cells by LDL-labeled PtdSer. Furthermore, IFN-γ activation of macrophages, in the absence of other pro-inflammatory stimuli, was shown to increase apoptotic uptake (135).

The ability of phagocytes to clear dead cells is also subject to regulation. This is the result of alterations in gene expression, which can function as negative feedback following initial engulfment of dying cells. For example, it has been shown that macrophages, upon engulfing apoptotic cells can undergo a form of activation and reprograming (136). As well as skewing the macrophage to a more anti-inflammatory phenotype, which in turn promotes inflammatory resolution, both mouse and human macrophages can upregulate CXCR4 during efferocytosis, which in turn encourages their recruitment to draining lymph nodes (137). These macrophages were also shown to subsequently reduce their efferocytosis capacity. Thus, apoptotic cells can reduce local levels of efferocytosis as well as promote them.

More recently macrophages were shown to regulate efferocytosis in surrounding non-circulating phagocytes, such as phagocytic airway epithelial cells (138). In response to IL-4 and IL-13, which are secreted by epithelia and stimulated Th2 cells, macrophages upregulated secretion of both insulin-like growth factor 1 (IGF-1) and microvesicles containing anti-inflammatory signals. Both microvesicles and IGF-1, in turn, fed back to epithelia, causing a reduction of apoptotic cell clearance in favor of microvesicle uptake.

In the context of the liver, some of the mechanisms described for the regulation of efferocytosis apply to circulating and resident macrophages/monocytes. Further, the neuronal guidance protein netrin-1 has been shown to promote resolution of ischemia/reperfusion injury, in part by increasing the capacity of Kupffer cells to engulf apoptotic cells (139). The same molecule was shown to promote liver regeneration (139). In a mouse model of colon carcinoma metastasis in the liver, intercellular cell adhesion molecule 1-deficient macrophages cocultured with tumor cells showed increased efferocytosis dependent on phosphatidylinositol 3 kinase (140).

Environmental factors can also affect phagocytosis, and this extends to the clearance of dead cells; studies in human skin have demonstrated that ethanol can reduce phagocytic function (141), and there have been reports on increased phagocytosis in ethanol-fed rats, which was modulated by diet (142). Hepatocyte phagocytosis of apoptotic cells was decreased in ethanol-fed rats compared to controls, therefore the effects of ethanol on efferocytosis may be cell type-dependent (97). It is unclear whether professional phagocytes play a role in the regulation of efferocytosis by hepatocytes and liver endothelial cells.

## The Impact of Efferocytosis by Tissue Epithelia

Non-professional efferocytes are important throughout all developmental stages of an organism and can take over the clearance of apoptotic cells in the absence of professional phagocytes (143). This was confirmed in PU.1 knockout mice that lack macrophages, and the removal of apoptotic cells required for foot-limb development was instead performed by mesenchymal cells (144). Non-professional phagocytes therefore contribute to efferocytosis, even at the earliest stages of development.

Some of the best-studied phagocytic epithelia are bronchial and alveolar epithelial cells (138, 145, 146). Epithelial cells lining the respiratory tract make first contact with airborne allergens such as house dust mite antigens. Subsequent inflammatory stimuli, including the recruitment of basophils, mast cells and lymphocytes, result in epithelial cell injury. Lung epithelia clear their dying neighbors through PtdSer and Rac1-dependent mechanisms, which can be modified experimentally (146). As with macrophages, apoptotic cell clearance by lung epithelia induced anti-inflammatory cytokines such as IL-10 and TGF-β. Conditional Rac1 deletion in mouse lung epithelia resulted in an exacerbated immune response and greater epithelial damage. These studies demonstrated the efficiency and importance for lung epithelial cell efferocytosis in the regulation of lung inflammation (146, 147).

Retinal epithelial cell efferocytosis has also been well characterized (148–151). Light-sensing cells of the retina are frequently turned over *via* programmed-cell death, often succumbing to autophagy-associated death, called *autolysis* (152, 153). Dysregulation of autophagy in these cells has been frequently reported to increase retinal pigment cell death (152, 154). Although a normal part of age-related macular degeneration, failure to clear these dying cells can accelerate retinal damage. Together with professional phagocytes, retinal pigment cells are also charged with the removal of dead cells, in a manner dependent on MerTK.

Throughout the lifecycle of an organism, the removal of immature cells or those with high turnover is necessary to maintain tissue homeostasis. Intravital microscopy has revealed how hair follicles in mice regress through programmed cell death of hair-producing basal epithelial cells (155). Neighboring cells of the same type then clear apoptotic cells through mechanisms requiring TGF-β signaling. In response to kidney damage, epithelial cells recognize and engulf PtdSer-positive apoptotic cells *via* KIM-1 or TIM-1 (99). Colonic epithelial cells have also been shown to engulf their apoptotic neighbors, which aids in maintaining low levels of inflammation (156).

Studies in multiple progenitor types have recently identified their importance in efferocytosis. Skeletal muscle progenitors recognizing PtdSer on neighboring apoptotic cells, receive the signal to differentiate and fuse into multinuclear myofibers (157). Mesenchymal stem cells take their cues from bone marrow apoptotic cells *via* efferocytosis and undergo osteogenic differentiation (158). Chondrogenic progenitor cells display macrophage-like abilities in that they react to “find-me” signals from apoptotic cells (159), and non-motile chondrocytes also have a role in efferocytosis (160). As previously discussed, neuronal progenitors which apoptose following failure to complete neural circuits throughout neurogenesis, were recognized and cleared by other progenitor cells *via* Rac1 activation following ELMO-1 signaling (161). Of note, neuronal and hepatic epithelia can be derived from common progenitor cells.

Through its cardinal role in the neutralization of toxic substances, to its frequent influx and arresting of leukocytes, the liver has evolved to cope well with cell death (10, 162). Although hepatocytes are somewhat resistant to intrinsic apoptotic pathways (163–165), many death receptors are ubiquitously expressed throughout the liver, increasing their susceptibility to extrinsic apoptosis by exposure to pro-inflammatory cytokines such as TNF family molecules including TNF-related apoptosis-inducing ligand (TRAIL) (166–170). Clearance of apoptotic cells by macrophages is a pro-resolution process, however, liver-infiltrating macrophages and Kupffer cells can upregulate death ligands in the liver, including FasL, TNF-α, and TRAIL, increasing the rate of local hepatocyte death and the risk of further inflammation (171, 172). Acute injury such as ischemia and the resulting trauma from hypoxia/reoxygenation can also result in similar sudden increases in necrotic cell death (164, 165, 169). Furthermore, steatosis—accumulation of lipids associated with a multitude of fatty liver diseases—can cause wide hepatocyte cell death *via* lipoapoptosis induced by ER stress-mediated intrinsic pathways (173, 174). Ethanol-induced injury can also have an impact on receptor-mediated endocytosis by the ASGPR and efferocytosis (175–178).

Failure to clear dead cells from the parenchyma is accumulatively detrimental to the liver; clearance of necrotic cells—both primary and secondary, resulting from uncleared apoptotic cells—results in increase in pro-inflammatory cell influx and cytokine secretion, leading to further damage to the liver (14). HMGB1 is important in liver protection from ischemia/reperfusion injury (179), yet in a sterile model it acted as a damage-associated molecular pattern that enhanced liver injury in both ischemia/reperfusion and POD models (180). Interactions between ASGPR on hepatocytes and B220 epitope of CD45 assist in the capture and trapping of apoptotic cells in the liver (96, 181, 182). The impact of hepatocyte efferocytosis on the inflammatory milieu remains to be established.

Beyond the capacity of hepatocytes for erythrocytosis (183), further evidence or insights into the mechanisms or anti-inflammatory impact of hepatocyte efferocytosis have not been elucidated. Hepatocytes express an array of immunomodulatory cytokines, including TNF-α and IL-10 (184–186); it is not known whether these are modulated during efferocytosis as in lung epithelia and in professional phagocytes. Understanding the molecular mechanisms, purpose and regulation of dead cell clearance by hepatocytes is vital to estimate its impact on the onset and resolution of inflammation, as elevation in hepatocyte apoptosis is key to the pathogenesis and progression of most forms of liver disease (14). Outstanding questions on hepatocyte efferocytosis include:

– *What are the molecules that mediate recognition and engulfment of apoptotic and/or necrotic cells by hepatocytes?* ASGPR is thus far the only receptor restricted to hepatocyte efferocytosis; despite its multiple roles in receptor-mediated efferocytosis, ASGPR-deficient mice develop normally yet have exacerbated pathology in liver injury models (177, 187).
– *Is efferocytosis by hepatocytes in portal and centrilobular regions mediated by the same molecular mechanisms?* These regions have differential access to apoptotic and necrotic cells, respectively, as well as nutrient, inflammatory infiltrate and oxygenation levels that may all influence the capacity for efferocytosis.
– How is hepatocyte efferocytosis regulated in health, infection, inflammation and cancer?
– Can hepatocyte efferocytosis be modulated by pharmacological interventions?
– Does efferocytosis affect the ability of hepatocytes to regenerate during injury?

## Clinical Implications of Defects in Efferocytosis

Failure to remove dying cells, both apoptotic and necrotic, have been connected to disease exacerbation (49). Accumulation of dying cells increases the availability of proimmunogenic factors and can increase the risk of autoimmunity, especially as death-recognition becomes skewed to proinflammatory recognition of secondary-necrotic cells. This topic was explored in a recent special issue in Frontiers in Immunology (188).

Defects in efferocytosis have also been shown to be beneficial for the longevity of tumors. Upregulation in the “don’t-eat me” signal CD47 was reported in myeloid leukemia (189, 190) which was associated with increased tumor survival and poorer prognosis. Similar pathogenic consequences of aberrant efferocytosis have been exemplified through deficiencies in death receptors (1). Loss of axl, MerTK, and its associated ligand, Gas6, have all been shown to promote the growth of colon cancers (191, 192). Conversely, loss of stabilin-1 has shown to reduce growth of implanted tumors in knockout mice, due to reduced recruitment of tumor-associated lymphocytes and macrophages (193). As such, loss of death-receptor expression is not always beneficial for cancer vitality. However, loss of other receptors for dying cells has displayed varying phenotypes associated with the lack of apoptotic cell clearance. Loss of SCARF1 and axl has been reported to promote autoimmunity (43, 194).

Similar dangers to those mentioned above regarding deficiencies in dying cell clearance are apparent for many liver diseases. In the context of the liver, the effects of efferocytosis in autoimmune family disorders have not been established directly. Reports on efferocytosis in liver diseases are listed in Table 2. Clearance of dying cells in the liver is thought to reduce the risk of autoimmune hepatitis and promote reversal of fibrosis by macrophages (195, 196). In primary biliary cholangitis, efferocytosis by biliary epithelia may be important in defining the tissue specificity of the autoimmune response (8, 197). It is worth considering that standard of care treatments for autoimmune conditions include corticosteroid regimens, which have been shown to upregulate efferocytosis (49, 198). Prevention of efferocytosis may therefore exacerbate liver diseases.

**Table 2:** The role of efferocytosis in liver diseases.

Liver disease	Efferocytosis relevance	Reference
Autoimmune hepatitis	Hepatocyte stress and correlations to disease	(39, 121, 195)
	Autoantibodies targeting ASGPR	

Primary biliary cholangitis	Phagocytes were shown to contain PDC-E2 immunogen	(8, 196, 199–201)
	Biliary injury clearance is linked to autoimmunity	
	Biliary injury clearance alleviates liver fibrosis	

Primary sclerosing cholangitis	Collection of genome-wide studies that show a role of apoptosis	(202)

Alcohol injury	Ethanol exacerbates injury in ASGPR-deficient model	(97, 177, 178, 203, 204)

Fatty liver diseases	The role of specialized proresolving mediators in obese individuals (enhance efferocytosis)	(205–207)

Other liver injuries	Alpha 1 antitrypsin rescues macrophage efferocytosis	(139, 196, 208)
	Netrin 1 rescues efferocytosis in murine I/R injury model	
	Efferocytosis and tissue remodeling in rat bile duct ligation model	

*Although efferocytosis is critical for liver homeostasis, there is limited information on specific efferocytosis pathways that contribute to liver disease pathogeneses. The importance of dead cell clearance is better established than the mechanisms that mediate efferocytosis in the inflamed or injured liver*.

As well as causing hepatocyte necrosis, chronic alcohol exposure was reported to reduce macrophage efferocytosis through diminishing MFG-E8 expression (209). Prevention of efferocytosis by macrophages in the liver could increase further inflammatory stimuli, although it is not clear how hepatocyte efferocytosis would be affected. Contrarily, reduced efferocytosis in certain disease models has been shown to be beneficial. Loss of the dead-cell receptor TIM4, for example, in a mouse model of ischemia/reperfusion injury reduced immune cell infiltration and hepatocyte damage (210). Understanding the protein-specific and situational benefits or detriments to reduced efferocytosis in diseases of the liver and other organs can give insights into possible therapeutics for tissue damage and autoimmunity.

## Conclusion

Recent advances in epithelial cell efferocytosis have highlighted the importance of tissue epithelia in the everyday clearance of billions of apoptotic cells. Compared to professional efferocytes, there is little known regarding the receptors and molecular processes involved in the recognition of apoptotic and necrotic cells by non-professional phagocytes, including molecules that may confer tissue-specific function. Given the impact of efferocytosis on the pathogenesis of autoimmunity, tissue injury and tumor biology (211), molecules driving efficient clearance of dead cells are valid therapeutic targets. Hepatocyte efferocytosis, accomplished at least in part by the liver-restricted ASGPR, is an attractive target for therapeutic intervention for a multitude of liver diseases.

## Ethics Statement

This study was carried out in accordance with the recommendations of LREC 06/Q2708/11, South Birmingham, Birmingham, UK. All subjects gave written informed consent in accordance with the Declaration of Helsinki.

## Author Contributions

SPD, GMR, and ZS performed stains for immunohistochemistry and immunofluorescence for illustrative purposes, and wrote the manuscript.

## Disclaimer

This article/paper/report presents independent research funded by the NIHR Birmingham Biomedical Research Centre at the University Hospitals Birmingham NHS Foundation Trust and the University of Birmingham. The views expressed are those of the author(s) and not necessarily those of the NHS, the NIHR or the Department of Health.

## Conflict of Interest Statement

The authors declare that the research was conducted in the absence of any commercial or financial relationships that could be construed as a potential conflict of interest.
